# Supplementary material for: Predictive Model and Risk Factors for Case Fatality of COVID-19: A Cohort of 21,392 Cases in Hubei, China
Source: Innovation (Camb). 2020 Aug 3;1(2):100022. doi: 10.1016/j.xinn.2020.100022 (PMC7832941; doi:10.1016/j.xinn.2020.100022)
Supplement: Document S1. Supplemental Experimental Procedures, Figures S1–S12, and Tables S1–S7 [file mmc1.pdf]

XINN, Volume *1*

Supplemental Information

Predictive Model and Risk Factors for Case Fatality of COVID-19: A Cohort of 21,392 Cases in Hubei, China

Ran Wu, Siqi Ai, Jing Cai, Shiyu Zhang, Zhengmin (Min) Qian, Yunquan Zhang, Yinglin Wu, Lan Chen, Fei Tian, Huan Li, Mingyan Li, and Hualiang Lin

Supplementary Materials

Table S1. Point assignment and one-, two- three- and four-week death probability of a male patient.

Comorbidity	Age group	Severity	Total points	Death probability (%)			
				Week 1	Week 2	Week 3	Week 4
No	< 45	Mild	163	1	1	2	3
		Moderate	166	1	1	2	3
		Severe	116	1	2	3	4
		Critical	66	2	5	7	13
	45-60	Mild	145	1	1	2	3
		Moderate	148	1	1	2	3
		Severe	98	2	4	4	5
		Critical	48	4	10	15	20
	≥ 60	Mild	110	1	2	3	4
		Moderate	113	1	2	3	4
		Severe	63	2	7	9	13
		Critical	13	15	26	36	44
Yes	< 45	Mild	150	1	1	2	3
		Moderate	153	1	1	2	3
		Severe	103	2	3	4	5
		Critical	53	3	8	13	16
	45-60	Mild	132	1	1	2	3
		Moderate	135	1	1	2	3
		Severe	85	1	3	4	6
		Critical	35	7	15	21	27
	≥ 60	Mild	97	2	4	4	5
		Moderate	100	2	4	4	5

		Severe	50	4	9	15	20
		Critical	0	21	36	46	54

Table S2. Point assignment and one-, two- three- and four-week death probability of a female patient.

**Comorbidity**	**Age group**	**Severity**	**Total points**	**Death probability (%)**			
				**Week 1**	**Week 2**	**Week 3**	**Week 4**
No	< 45	Mild	173	1	1	2	3
		Moderate	176	1	1	2	3
		Severe	126	1	1	2	3
		Critical	76	2	4	7	8
	45-60	Mild	155	1	1	2	3
		Moderate	158	1	1	2	3
		Severe	108	2	4	3	4
		Critical	58	3	8	11	15
	≥ 60	Mild	120	1	2	3	4
		Moderate	123	1	2	3	4
		Severe	73	2	5	8	9
		Critical	23	11	20	30	36
Yes	< 45	Mild	160	1	1	2	3
		Moderate	163	1	1	2	3
		Severe	113	2	3	4	5
		Critical	63	2	6	10	14
	45-60	Mild	142	1	1	2	3
		Moderate	145	1	1	2	3
		Severe	95	2	4	5	5
		Critical	45	5	11	16	22

≥ 60	Mild	107	2	4	4	5
	Moderate	110	2	4	4	5
	Severe	60	3	6	10	15
	Critical	10	16	30	40	46

Table S3. The comparison of general characteristics of the included (n=21,392) and excluded participants (n=48,841) in Hubei, China.

**Variable**	**Included (n=21,392)**	**Excluded (n=48,841)**	***P* value**
**Sex**			<0.05
Male	11,099 (51.88)	23,295 (47.69)	
Female	10,293 (48.12)	25,548 (52.31)	
**Age**			<0.05
< 45 years	8,127 (37.99)	14,445 (29.57)	
45-60 years	7,641 (35.72)	15,046 (30.80)	
≥ 60 years	5,624 (26.29)	19,352 (39.62)	
**Death**			0.21
Deceased	1,020 (4.77)	2,441 (5.00)	
Alive	20,372 (95.23)	46,402 (95.00)	

Table S4. Sensitivity analysis by restricting the participants to the confirmed cases (n=18,470).

	**Univariable model**		**Multivariable model**	
	**HR (95% CI)**	***P* value**	**HR (95% CI)**	***P* value**
**Sex**				
Female	1.00		1.00	
Male	1.67 (1.46, 1.92)	<0.001	1.34 (1.17, 1.53)	<0.01

**Age group**				
< 45 years	1.00		1.00	
45-60 years	3.26 (2.37, 4.48)	<0.001	2.28 (1.65, 3.15)	<0.001
≥ 60 years	21.98 (16.50, 29.27)	<0.001	7.04 (5.14, 9.62)	<0.001
**Comorbidity**				
No	1.00		1.00	
Yes	4.01 (3.52, 4.56)	<0.001	1.43 (1.25, 1.64)	<0.001
**Severity**				
Mild/asymptomatic	1.00		1.00	
Moderate	1.04 (0.73, 1.47)	>0.05	1.08 (0.76, 1.53)	>0.05
Severe	12.43 (9.36, 16.51)	<0.001	7.86 (5.90, 10.47)	<0.001
Critical	76.75 (58.50, 100.69)	<0.001	42.57 (32.26, 56.19)	<0.001

Table S5. Comparison of the effects of the general demographic factors using the included (n=21,392) and excluded participants (n=48,841).

	**Included participants**		**Excluded participants**	
	**HR (95% CI)**	***P* value**	**HR (95% CI)**	***P* value**
**Sex**				
Female	1.00		1.00	
Male	1.72 (1.51, 1.95)	<0.001	1.91 (1.76, 2.08)	<0.001
**Age group**				
< 45 years	1.00		1.00	
45-60 years	3.56 (2.62, 4.83)	<0.001	4.27 (3.34, 5.46)	<0.001
≥ 60 years	21.36 (16.20, 28.16)	<0.001	19.27 (15.34, 24.19)	<0.001
**Residence**				
Local	1.00		1.00	
Migrant	1.11 (0.96, 1.28)	>0.05	1.33(1.23, 1.45)	<0.001

**Area**				
Hubei-other cities	1.00		1.00	
Hubei-Wuhan	1.53 (1.44, 1.62)		1.62 (1.49, 1.76)	
**Period**				
Before Feb. 8	1.00		1.00	
After Feb. 8	0.44 (0.37, 0.52)	<0.001	0.53 (0.49, 0.57)	<0.001

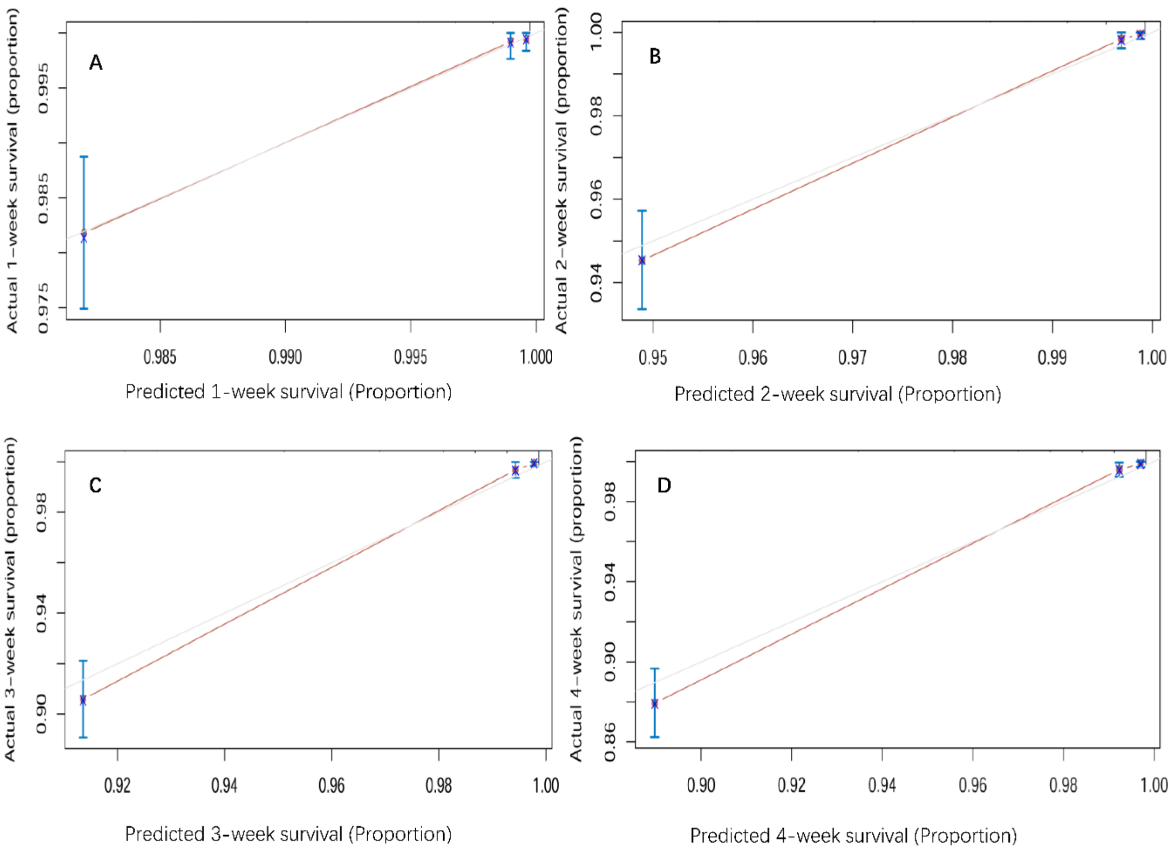

Figure S1. The calibration curve for predicting patient survival in (A) one-week and, (B) two-week, (C) three-week and (D) four-week in the validation cohort.

Note: Nomogram-predicted survival probability of patients with COVID-19 is plotted on the x-axis; actual survival probability is plotted on the y-axis.
