# Supplementary material for: Predictive Model and Risk Factors for Case Fatality of COVID-19: A Cohort of 21,392 Cases in Hubei, China
Source: Innovation (Camb). 2020 Aug 3;1(2):100022. doi: 10.1016/j.xinn.2020.100022 (PMC7832941; doi:10.1016/j.xinn.2020.100022)
Supplement: Document S2. Article plus Supplemental Information [file mmc2.pdf]

Article

Predictive Model and Risk Factors for Case Fatality of COVID-19: A Cohort of 21,392 Cases in Hubei, China

Ran Wu,[1,5] Siqi Ai,[2,5] Jing Cai,[1] Shiyu Zhang,[2] Zhengmin (Min) Qian,[3] Yunquan Zhang,[4] Yinglin Wu,[2] Lan Chen,[2] Fei Tian,[2] Huan Li,[2] Mingyan Li,[1,*] and Hualiang Lin[2,6,*]

[1]Institute of Preventive Medicine Information, Hubei Provincial Center for Disease Control and Prevention, 6 Zhuodaoquan North Road, Wuhan, Hubei 430079, China
[2]Department of Epidemiology, School of Public Health, Sun Yat-sen University, 74 Zhongshan 2nd Road, Guangzhou, Guangdong 510080, China
[3]College for Public Health & Social Justice, Saint Louis University, St. Louis, MO, USA
[4]Department of Epidemiology and Biostatistics, School of Public Health, Medical College, Wuhan University of Science and Technology, Wuhan 430065, China
[5]These authors contributed equally
[6]Lead Contact
*Correspondence: hbcdc_limingyan@163.com (M.L.); linhualiang@mail.sysu.edu.cn (H.L.)
Received: April 15, 2020; Accepted: July 21, 2020; Published: August 28, 2020; https://doi.org/10.1016/j.xinn.2020.100022
© 2020 The Author(s).

PUBLIC SUMMARY

- 21,392 COVID-19 patients constituted one of the largest cohort studies to date
- Elderly male patients with critical illness and comorbidities had higher death rate
- The death probability increased with time, which was evident for critically ill patients
- The highest death probability within 1 month can reach 54% by the predictive model
- The predictive model could guide the allocation of medical resources

GRAPHICAL ABSTRACT

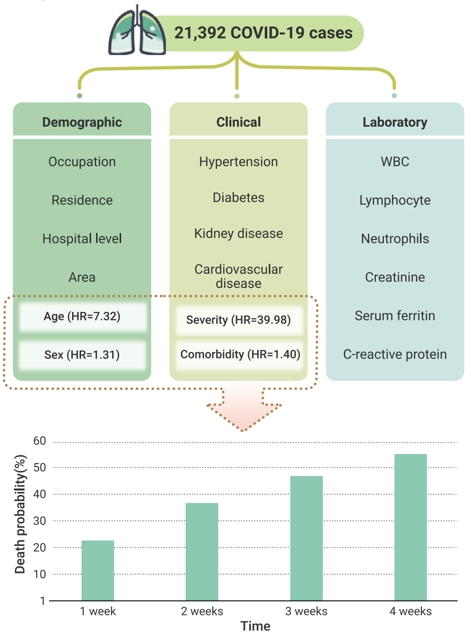

CORRESPONDENCE

hbcdc_limingyan@163.com (M.L.); linhualiang@mail.sysu.edu.cn (H.L.)

https://doi.org/10.1016/j.xinn.2020.100022

Received: April 15, 2020
Accepted: July 21, 2020
Published: August 28, 2020

www.cell.com/the-innovation

Article

Predictive Model and Risk Factors for Case Fatality of COVID-19: A Cohort of 21,392 Cases in Hubei, China

Ran Wu,[1,5] Siqi Ai,[2,5] Jing Cai,[1] Shiyu Zhang,[2] Zhengmin (Min) Qian,[3] Yunquan Zhang,[4] Yinglin Wu,[2] Lan Chen,[2] Fei Tian,[2] Huan Li,[2] Mingyan Li,[1,]* and Hualiang Lin[2,6,]*

[1]Institute of Preventive Medicine Information, Hubei Provincial Center for Disease Control and Prevention, 6 Zhuodaoquan North Road, Wuhan, Hubei 430079, China

[2]Department of Epidemiology, School of Public Health, Sun Yat-sen University, 74 Zhongshan 2nd Road, Guangzhou, Guangdong 510080, China

[3]College for Public Health & Social Justice, Saint Louis University, St. Louis, MO, USA

[4]Department of Epidemiology and Biostatistics, School of Public Health, Medical College, Wuhan University of Science and Technology, Wuhan 430065, China

[5]These authors contributed equally

[6]Lead Contact

*Correspondence: hbcdc_limingyan@163.com (M.L.); linhualiang@mail.sysu.edu.cn (H.L.)

Received: April 15, 2020; Accepted: July 21, 2020; Published: August 28, 2020; https://doi.org/10.1016/j.xinn.2020.100022

© 2020 The Author(s). This is an open access article under the CC BY license (http://creativecommons.org/licenses/by/4.0/).

An increasing number of patients are being killed by coronavirus disease 2019 (COVID-19), however, risk factors for the fatality of COVID-19 remain unclear. A total of 21,392 COVID-19 cases were recruited in the Hubei Province of China between December 2019 and February 2020, and followed up until March 18, 2020. We adopted Cox regression models to investigate the risk factors for case fatality and predicted the death probability under specific combinations of key predictors. Among the 21,392 patients, 1,020 (4.77%) died of COVID-19. Multivariable analyses showed that factors, including age (≥60 versus <45 years, hazard ratio [HR] = 7.32; 95% confidence interval [CI], 5.42, 9.89), sex (male versus female, HR = 1.31; 95% CI, 1.15, 1.50), severity of the disease (critical versus mild, HR = 39.98; 95% CI, 29.52, 48.86), comorbidity (HR = 1.40; 95% CI, 1.23, 1.60), highest body temperature (>39°C versus <39°C, HR = 1.28; 95% CI, 1.09, 1.49), white blood cell counts (>10 × 10^9/L versus (4–10) × 10^9/L, HR = 1.69; 95% CI, 1.35, 2.13), and lymphocyte counts (<0.8 × 10^9/L versus (0.8–4) × 10^9/L, HR = 1.26; 95% CI, 1.06, 1.50) were significantly associated with case fatality of COVID-19 patients. Individuals of an older age, who were male, with comorbidities, and had a critical illness had the highest death probability, with 21%, 36%, 46%, and 54% within 1–4 weeks after the symptom onset. Risk factors, including demographic characteristics, clinical symptoms, and laboratory factors were confirmed to be important determinants of fatality of COVID-19. Our predictive model can provide scientific evidence for a more rational, evidence-driven allocation of scarce medical resources to reduce the fatality of COVID-19.

KEYWORDS: COVID-19; FATALITY; RISK FACTOR

INTRODUCTION

An unprecedented outbreak of coronavirus disease (COVID-19) has swept across the globe since the end of 2019.[1,2] As of April 28, 2020, 3,034,801 confirmed COVID-19 cases, and 210,511 deaths have been reported globally, with an overall case fatality rate (CFR) of 6.9%.

The CFR of COVID-19 varied greatly across countries and geographical areas.[3,4] The exploration of risk factors which contributed to the variation in CFR of COVID-19 has significant clinical significance, since this effort could help identify vulnerable patients who most need clinical treatment and care. However, the risk factors for its case fatality have been only examined in a few studies with a relatively small sample size in Wuhan, China.[5–9] The information is urgently needed for countries where it is not feasible to treat every patient due to limited medical resources and capacity. In this regard, if we identify the most vulnerable subgroups among the increasing number of cases, the limited medical resources can be rationally targeted to this group to reduce the fatality rate.[10] In addition, no studies have explored the survival of COVID-19 patients under different combinations of important determinants of case fatality.

We thus conducted this study with the aim to identify the risk factors for COVID-19 case fatality using the data of 21,392 patients from Hubei Province. We further constructed a predictive model based on the estimated parameters from Cox analyses, which could be used in clinical decision-making.

RESULTS

Descriptive Results

As of February 27, 2020, 70,233 COVID-19 cases were reported in Hubei Province, including confirmed, clinical, suspected, and asymptomatic cases, some of which were excluded from diagnosis during follow-up. Among them, 21,392 cases were selected for our interview and included in this study. Table S3 shows the comparison of the general demographic characteristics of the included and excluded cases; the two groups had a similar fatality rate (4.77% versus 5.00%, p = 0.21). However, the included participants were more likely to be males and be of a young age. There was no loss to follow-up during the follow-up period, and 1,020 patients died of COVID-19, giving an overall CFR of 4.77% in Hubei. The CFR across different characteristics is presented in Table 1. Patients aged 60 years and older presented the highest fatality rate (14.05%) compared with other groups. A relatively higher fatality rate was observed among males (5.87%) than females (3.58%). Patients with comorbidities had a higher fatality rate (11.33%) than those without (2.98%). An increasing fatality rate was found with higher disease severity, with the highest in the critical level (48.59%). The patients with a longer interval between symptom onset and diagnosis had a higher fatality rate (7.87% for those longer than 14 days and 4.27% for those fewer than 7 days). Patients admitted to hospital before February 8 had a higher fatality rate (5.97%) than those admitted after February 8, 2020 (2.41%). Relatively higher fatality rates were also present among patients with higher WBC counts (16.50%), a lower count (8.86%) and percentage (8.59%) of lymphocytes, and a higher percentage of neutrophils (7.93%). Differential CFRs were also found across different strata of other factors, such as level of admitted hospital, occupation, and residence.

Risk Factors of Fatality of COVID-19

Table 2 shows the associations between potential risk factors and fatality risk from COVID-19. In the univariate analyses, age, sex, longer intervals between symptom onset and diagnosis, non-medical-related occupation, migrants, admittance before February 8, admittance to a higher level of hospital, severe and critical status, and living in Wuhan city were significantly associated with a higher fatality rate. In multivariate analyses, we observed an increased case fatality among patients aged 45–60 years (HR = 2.35; 95%

Table 1. Descriptive Characteristics of Study Participants by Survival Status in Hubei, China

Variable	Deceased, n (%)	Alive, n (%)	p Value
Sex			<0.01
Male	652 (5.87)	10,447 (94.13)	
Female	368 (3.58)	9,925 (96.42)	
Age group			<0.001
<45 years	54 (0.66)	8,073 (99.34)	
45–60 years	176 (2.30)	7,465 (97.70)	
≥60 years	790 (14.05)	4,834 (85.95)	
Occupation			<0.001
Medical-related	12 (1.08)	1,101 (98.92)	
Service-related	7 (2.94)	231 (97.06)	
Office worker	100 (2.07)	4,733 (97.93)	
Home worker	810 (6.00)	12,697 (94.00)	
Others	91 (5.35)	1,610 (94.65)	
Residence			<0.01
Local	749 (4.20)	17,079 (95.80)	
Migrant	271 (7.60)	3,293 (92.40)	
Time between symptom onset and diagnosis			<0.001
<7 days	550 (4.27)	12,317 (95.73)	
7–14 days	350 (5.00)	6,650 (95.00)	
≥14 days	120 (7.87)	1,405 (92.13)	
Period			<0.001
Before February 8	846 (5.97)	13,319 (94.03)	
After February 8	174 (2.41)	7,073 (97.59)	
Hospital level			<0.001
Grade III	583 (6.43)	8,485 (93.57)	
Grade II	410 (3.81)	10,364 (96.19)	
Grade I	27 (1.74)	1,523 (98.26)	
Severity			<0.001
Mild/asymptomatic	72 (0.85)	8,440 (99.15)	
Moderate	75 (0.85)	8,721 (99.15)	
Severe	306 (10.49)	2,611 (89.51)	
Critical	567 (48.59)	600 (51.41)	
Live nearby a farmers' market			>0.05
Yes	221 (4.70)	4,482 (95.30)	
No	799 (4.79)	15,890 (95.21)	
Comorbidity			<0.001
Yes	520 (11.33)	4,071 (88.67)	
No	500 (2.98)	16,301 (97.02)	

Table 1. *Continued*

Variable	Deceased, n (%)	Alive, n (%)	p Value
WBC count (n = 12,948)			<0.001
$<4 \times 10^9$/L	137 (3.42)	3,865 (96.58)	
$4–10 \times 10^9$/L	350 (4.19)	7,996 (95.81)	
$>10 \times 10^9$/L	99 (16.50)	501 (83.50)	
Lymphocytes count (n = 12,872)			<0.001
$<0.8 \times 10^9$/L	251 (8.86)	2,583 (91.14)	
$0.8–4 \times 10^9$/L	295 (3.05)	9,385 (96.95)	
$>4 \times 10^9$/L	19 (5.31)	339 (94.69)	
Lymphocytes percentage (n = 12,924)			<0.001
<20%	385 (8.59)	4,076 (91.41)	
20%–40%	169 (2.41)	6,843 (97.59)	
>40%	34 (2.34)	1,417 (97.66)	
Neutrophils percentage (n = 12,925)			<0.001
<50%	54 (2.77)	1,894 (97.23)	
50%–70%	153 (2.45)	6,084 (97.55)	
>70%	376 (7.93)	4,364 (92.07)	

CI, 1.73, 3.21) and among the elderly aged 60 years and above (HR = 7.32; 95% CI, 5.42, 9.89). Males (HR = 1.31; 95% CI, 1.15, 1.50) and patients admitted before February 8 were more likely to die of COVID-19. Significantly higher fatality risks were found among patients with severe symptoms, especially for critical level (HR = 39.98; 95% CI, 29.52, 48.86). The fatality rate was higher among patients living in Wuhan with an HR of 2.61 (95% CI, 2.26, 3.01) compared with those from other cities of Hubei Province.

Table 3 demonstrates the association between comorbidity, clinical symptoms, laboratory tests, and death risk of COVID-19 patients. The HR of death was higher in patients with existing chronic diseases (such as hypertension, diabetes, and kidney disease) in both univariate and multivariate models. For example, existing hypertension (HR = 1.29; 95% CI, 1.13, 1.47), diabetes (HR = 1.27; 95% CI, 1.06, 1.51), and kidney disease (HR = 1.53; 95% CI, 1.13, 2.07) were all associated with increased risk of death. Regarding the clinical symptoms, we found that no dry cough, no muscular soreness, and the highest body temperature being higher than 39°C (HR = 1.28; 95% CI, 1.09, 1.49) was associated with a higher fatality risk. Regarding the laboratory testing, we found that WBC counts higher than 10×10^9/L (HR = 1.69; 95% CI, 1.35, 2.13), lymphocytes lower than 0.8×10^9/L (HR = 1.26; 95% CI, 1.06, 1.50), lymphocyte percentage below 20% (HR = 1.52; 95% CI, 1.26, 1.82), and neutrophil percentage above 70% (HR = 1.45; 95% CI, 1.20, 1.76) were also associated with a higher fatality risk from COVID-19.

Figure 1 shows the survival curves by sex, age, severity of illness, and comorbidity condition. The survival probability was remarkably higher in female participants than male patients and was lower among the older patients and patients with severe illnesses and existing chronic diseases.

Our sensitivity analysis based on confirmed cases produced similar results (Table S4). For example, the HR for older age (≥60 versus <45 years) was 7.04 (95% CI, 5.14, 9.62) in multivariate analysis, which is consistent with that of the original analysis (HR = 7.32; 95% CI, 5.42, 9.89). We analyzed the effects of demographic factors among the included and excluded participants, and found a generally comparable result among the two groups (Table S5). For example, the HR for male (versus female) was 1.72 (95% CI, 1.51, 1.95) among included patients and 1.91 (95% CI, 1.76, 2.08) among the excluded participants, indicating that the analysis based on the included participants may provide a representative result.

Table 2. Univariate and Multivariate Analysis of Demographic Characteristics and Basic Information Associated with Risk of Death from COVID-19

	Univariable Model		Multivariable Model	
	HR (95% CI)	p Value	HR (95% CI)	p Value
Sex				
Female	1.00		1.00	
Male	1.66 (1.46, 1.89)	<0.001	1.31 (1.15, 1.50)	<0.01
Age group				
<45 years	1.00		1.00	
45–60 years	3.41 (2.52, 4.63)	<0.001	2.35 (1.73, 3.21)	<0.001
≥60 years	22.44 (17.03, 29.56)	<0.001	7.32 (5.42, 9.89)	<0.001
Time between symptom onset and diagnosis				
<7 days	1.00		1.00	
7–14 days	1.06 (0.93, 1.21)	>0.05	0.69 (0.60, 0.79)	<0.001
≥14 days	1.59 (1.30, 1.94)	<0.001	0.54 (0.44, 0.66)	<0.001
Occupation				
Medical-related	1.00		1.00	
Service-related	2.86 (1.12, 7.25)	<0.05	3.07 (1.20, 7.85)	<0.01
Office worker	1.94 (1.06, 3.52)	<0.05	1.92 (1.05, 3.50)	<0.01
Home worker	5.73 (3.24, 10.13)	<0.001	1.76 (0.98, 3.16)	>0.05
Others	5.07 (2.78, 9.26)	<0.001	2.13 (1.16, 3.92)	<0.01
Residence				
Local	1.00		1.00	
Migrant	1.75 (1.52, 2.01)	<0.001	0.86 (0.74, 1.01)	>0.05
Period				
Before February 8	1.00		1.00	
After February 8	0.46 (0.39, 0.54)	<0.001	0.54 (0.46, 0.63)	<0.001
Hospital level				
Grade I	1.00		1.00	
Grade II	2.02 (1.37, 2.99)	<0.001	1.10 (0.74, 1.64)	>0.05
Grade III	3.39 (2.31, 4.99)	<0.001	1.15 (0.77, 1.73)	>0.05
Severity				
Mild/asymptomatic	1.00		1.00	
Moderate	0.94 (0.98, 1.30)	>0.05	0.97 (0.70, 1.33)	>0.05
Severe	11.97 (9.26, 15.48)	<0.001	7.05 (5.44, 9.16)	<0.001
Critical	70.65 (55.28, 90.34)	<0.001	39.98 (29.52, 48.86)	<0.001
Live nearby a farmers' market				
No	1.00		1.00	
Yes	0.94 (0.81, 1.09)	>0.05	0.75 (0.65, 0.88)	<0.001
Transfer to another hospital				
No	1.00		1.00	
Yes	1.03 (0.85, 1.23)	>0.05	0.70 (0.61, 0.82)	<0.001
Area				
Hubei-other cities	1.00		1.00	
Hubei-Wuhan	3.04 (2.69, 3.45)	<0.001	2.61 (2.26, 3.01)	<0.001

Prediction Model for Death Probability

The established nomogram demonstrated the contributions of each stratum within a specific variable to the probability of death (Figure 2). For example, given a patient who is an elderly male over 60 years without existing chronic diseases but with a severe status of COVID-19, the scores of corresponding Points for Age, Sex, Conditions, and Severity are 0, 0, 13, and 50, respectively. The total score is 63, which corresponds to a death probability of 2% within 1 week.

Tables S1 and S2 illustrate the contribution of sex, age, comorbidity, and severity to the probability of death. In general, with an older age and higher severity of illness, the death probability would be higher; the death probability of patients with comorbidity was higher than those without, and the death probability was higher for males than females. We found that an elderly patient over 60 years with comorbidity and critical illness had the highest death probability among different combinations, with 21%, 36%, 46%, and 54% within 1–4 weeks after the symptom onset, respectively. The estimated C-index was 0.93 (95% CI, 0.92, 0.93), indicating an ideal predictive accuracy of the model. The calibration curves suggested a good agreement in the validation cohort between the nomogram prediction and actual observation for 1-, 2-, 3-, and 4-week survival (Figure S1).

DISCUSSION

With data from 21,392 COVID-19 patient, we confirmed several important risk factors of fatality of COVID-19, including demographic factors, comorbidity, clinical symptoms, and laboratory findings. We further established a predictive model for the probability of death for COVID-19 patients using age, sex, severity of this disease, and comorbidity as the independent predictors. Findings from this study will provide important implications for the appropriate treatment of the COVID-19 cases in a more rational way worldwide.

Consistent with previous studies, our study found that age and sex were important independent predictors of fatality risk of COVID-19 patients.[5,11–13] For instance, one study, including 191 COVID-19 patients found that older age was positively associated with an increased risk of in-hospital death with an odds ratio (OR) of 1.10 (95% CI, 1.03, 1.17) for each year increase.[5] Similarly, Wu et al.[11] found that older age was associated with an increased risk of developing ARDS and subsequent death, probably due to the relatively lower immune function in elderly patients. Older individuals were believed to be physically weak and were more likely to have some comorbidities, which resulted in the higher risk of adverse outcomes, even death.[10] Jin et al.[13] analyzed the distribution of sex among a dataset that included 1,019 survival patients and 37 deaths due to COVID-19 and found that males tended to have more severe illnesses than females. In this study, a higher fatality rate was found among males than females (5.87% versus 3.58%), indicating that males may be more prone to greater disease severity and mortality.[13] The underlying mechanisms about the differential prognosis between males and females might be related to ACE2 receptor-mediated cell damage. Similar with SARS-CoV, the clinical deterioration of in patients with SARS-CoV-2 might result from an immune response induced by an inflammatory storm and cytopathic effects, which may cause severe ARDS.[14,15]

Table 3. Univariable and Multivariable Analyses for Comorbidities, Clinical Symptoms, and Laboratory Findings Associated with Case Fatality from COVID-19

	Univariable Model		Multivariable Model	
	HR (95% CI)	p Value	HR (95% CI)	p Value
With comorbidity				
No	1.00		1.00	
Yes	3.93 (3.47, 4.44)	<0.001	1.40 (1.23, 1.60)	<0.001
Hypertension				
No	1.00		1.00	
Yes	3.72 (3.27, 4.23)	<0.001	1.29 (1.13, 1.47)	<0.001
Diabetes				
No	1.00		1.00	
Yes	3.30 (2.78, 3.92)	<0.001	1.27 (1.06, 1.51)	<0.01
Kidney disease				
No	1.00		1.00	
Yes	4.33 (3.20, 5.86)	<0.001	1.53 (1.13, 2.07)	<0.01
Liver disease				
No	1.00		1.00	
Yes	1.07 (0.51, 2.25)	>0.05	1.08 (0.51, 2.27)	>0.05
Cardiovascular disease				
No	1.00		1.00	
Yes	4.97 (4.24, 5.83)	<0.001	1.51 (1.28, 1.77)	<0.001
Respiratory disease				
No	1.00		1.00	
Yes	2.07 (1.60, 2.67)	<0.001	1.23 (0.95, 1.59)	>0.05
Immune system disease				
No	1.00		1.00	
Yes	0.94 (0.13, 6.64)	>0.05	0.84 (0.12, 5.99)	>0.05
**Clinical Symptoms**				
Fever				
No	1.00		1.00	
Yes	1.26 (1.07, 1.47)	<0.01	1.03 (0.88, 1.21)	>0.05
Dry cough				
No	1.00		1.00	
Yes	0.89 (0.78, 1.01)	>0.05	0.87 (0.76, 1.00)	<0.05
Fatigue				
No	1.00		1.00	
Yes	1.24 (1.09, 1.42)	<0.001	0.95 (0.853, 1.08)	>0.05
Dyspnea				
No	1.00		1.00	
Yes	2.00 (1.75, 2.27)	<0.001	1.10 (0.96, 1.25)	<0.10
Muscular soreness				
No	1.00		1.00	
Yes	0.78 (0.64, 0.94)	<0.01	0.68 (0.56, 0.83)	<0.01
Highest body temperature				
<39°C	1.00		1.00	
>39°C	1.91 (1.65, 2.23)	<0.001	1.28 (1.09, 1.49)	<0.001
**Laboratory Testing**				
WBC count				
4–10 × 10^9/L	1.00		1.00	
<4 × 10^9/L	0.79 (0.65, 0.96)	<0.05	0.83 (0.68, 1.01)	>0.05
>10 × 10^9/L	4.24 (3.39, 5.30)	<0.001	1.69 (1.35, 2.13)	<0.001
Lymphocytes				
0.8–4 × 10^9/L	1.00		1.00	
<0.8 × 10^9/L	2.91 (2.46, 3.44)	<0.001	1.26 (1.06, 1.50)	<0.01
>4 × 10^9/L	1.74 (1.09, 2.77)	<0.05	1.23 (0.77, 1.97)	>0.05
Lymphocytes				
20%–40%	1.00		1.00	
<20%	3.61 (3.01, 4.33)	<0.001	1.52 (1.26, 1.82)	<0.001
>40%	0.98 (0.68, 1.41)	>0.05	1.14 (0.79, 1.66)	>0.05
Neutrophils				
50%–70%	1.00		1.00	
<50%	1.13 (0.83, 1.54)	>0.05	1.17 (0.86, 1.61)	>0.05
>70%	3.26 (2.70, 3.94)	<0.001	1.45 (1.20, 1.76)	<0.001

An animal experiment suggested that sex-dependent differences in disease severity stemmed from the enhanced susceptibility of male mice and lack of protective effect from the sex-based estrogen receptor.[16]

We found that the severe and critical status of illness were significantly associated with increased fatality risks. This finding was consistent with the study by Chen et al.,[17] which indicated that most patients with severe acute respiratory distress syndrome worsened in a short period of time and died of multiple organ failure. Given that severe patients were more likely to be elderly, obese, have comorbidities,[5,12,18] and suffer from organ and coagulation dysfunction (e.g., higher lactate dehydrogenase)[11] compared with non-severe patients, the implementation of early identification of patients with severe illness at an early stage was important for the management and treatment of patients with COVID-19.

Existing comorbidity was found to be associated with increased fatality risk of COVID-19 in our study, which was consistent with a few previous studies. For example, Huang et al.[19] found that a known history of type 2 diabetes may increase the likelihood of developing severe illness in COVID-19 patients by six times. Another study demonstrated that 64.3% of the patients had at least one comorbidity, the most common of which were hypertension (30%) and diabetes (12.1%), and older males with comorbidities were more likely to be affected by COVID-19.[20]

The observed protective effect of muscular soreness was beyond our expectation, but the underlying reason for this finding was not clear. We speculate that it might be because the clinical identification of muscular soreness

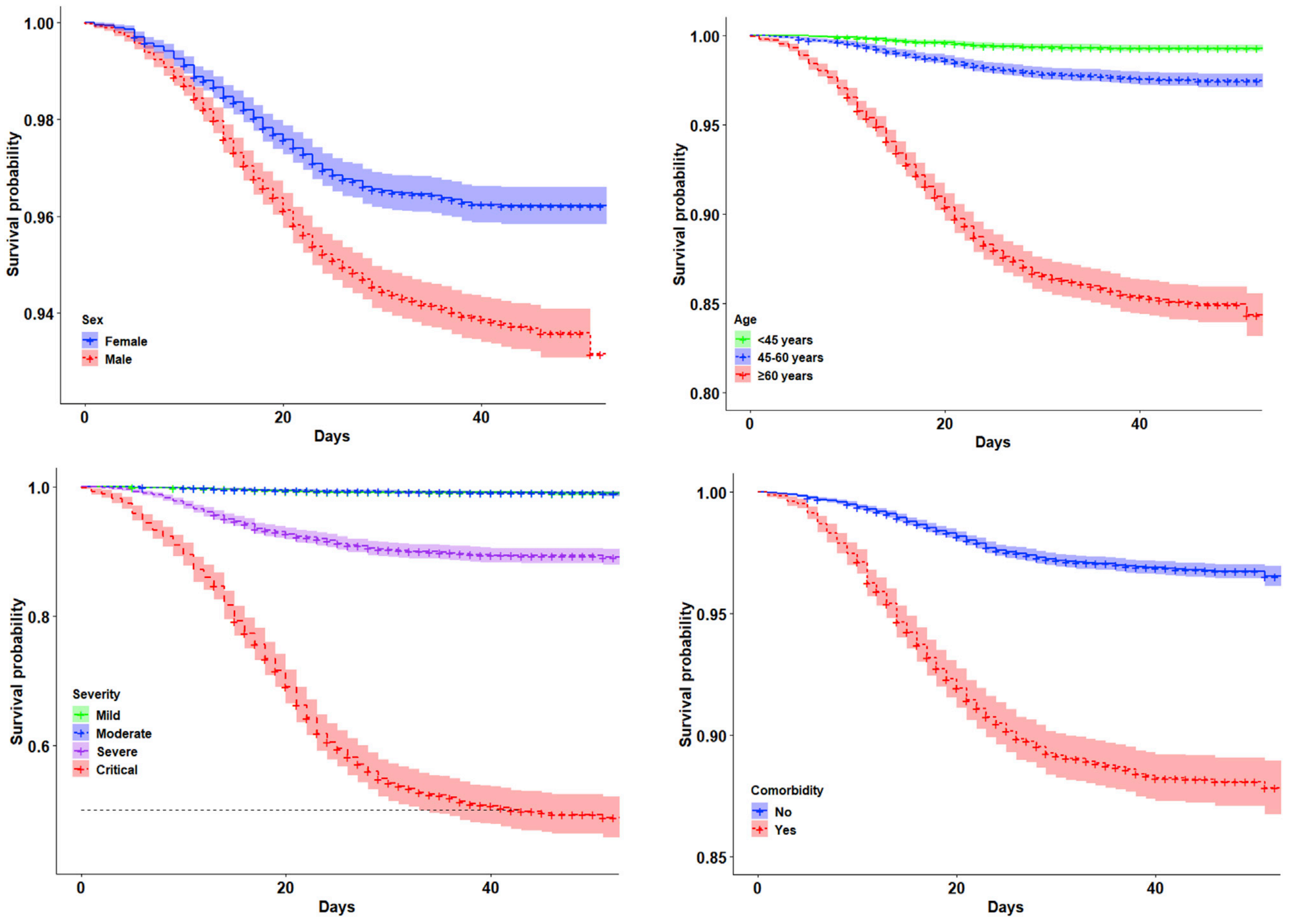

Figure 1. Survival Curves of COVID-19 Cases. The survival curves showed probability of survival in patients with regards to sex (top left), age (top right), severity (bottom left), and comorbidity (bottom right). The vertical axis represents the survival probability of an individual, the horizontal axis represents the days of survival. A score was assigned to each stratum within these variables on the point scale, and a straight line was drawn down to estimate the death probability at different time point by summing up the separate score and locating it on the Total Points axis.

was subjective. It is possible that different clinical doctors might have different criteria for the diagnosis of this symptom, so this finding should be interpreted cautiously.

Laboratory tests suggested that higher WBC counts, lower lymphocyte counts, and higher neutrophil counts may be risk factors of the risk of fatality. Several laboratory studies on immune cells supported this finding.[11,12,18] Based on the pathological process of SARS-CoV-2,[1] a high stimulus of inflammatory cytokines resulted in leukocytosis, and then a high degree of apoptosis of lymphocytes led to lymphopenia and neutrophilia. Recently, it has been reported that SARS-CoV-2 could enter the cells through the angiotensin converting enzyme 2 (ACE2) and then attack multiple organs and trigger fatal complications.[21,22]

Admission after February 8 was a protective factor for death compared with admission before February 8. It may be due to a scarcity of knowledge and understanding of the infection at the early stage as well as a lack of medical resources, which were largely improved by subsequent measures, including the establishment of more hospitals and the dispatch of medical experts and medical supplies from the national and provincial level.

It was beyond our expectation that a shorter time interval from symptom onset to diagnosis, being admitted to higher levels of hospital, and patients in Wuhan were associated with a higher fatality risk in this study. It was possible that patients with a longer interval between symptom onset and diagnosis were more likely to be the mild cases, and the patients admitted to the higher levels of hospitals had a severe illness.[23] Although, when compared with other cities in this province Wuhan is believed to have a higher level of medical care, during the outbreak of the disease there was such a serious shortage of medical resource and adverse medical environment[24] that some patients were unable to get timely medical treatment. These patients thus had a higher fatality risk. In fact, one study reported a 7-fold higher fatality rate of patients in Hubei Province compared with areas outside of this region, emphasizing the importance of strengthening the regulatory capacity of health systems in caring for critically ill patients with COVID-19.[25]

The severity of the disease was found to play a critical role in disease progression, followed by age. The individuals prone to be affected were found to be elderly men over 60 years with a critical stage of the illness and previous comorbidities. These observations were in agreement with previous studies. For example, Zhou et al.[5] conducted a retrospective cohort study on adult inpatients in two hospitals in Wuhan, China, and found that older age (OR = 1.10; 95% CI, 1.03, 1.17), blood levels of D-dimer (OR = 18.42; 95% CI, 2.64, 128.55), and sequential organ failure assessment scores (OR = 5.65; 95% CI, 2.61, 12.23) were associated with increased risk of in-hospital death. Wu et al.[11] investigated 201 COVID-19 cases using a Cox model, and found that risk factors associated with the fatality of COVID-19 were older age (HR = 6.17; 95% CI, 3.26, 11.67), neutrophilia (HR = 1.08; 95% CI, 1.01, 1.17), and organ and coagulation dysfunction (HR = 1.30; 95% CI, 1.11, 1.52). We went one step further than previous studies and established a model to predict the probability of death for patients with pneumonia between 1 and 4 weeks after diagnosis. The rapid increased probability of death (up to 0.48 and 0.55, corresponding to 3 and 4 weeks) indicated that illness progression in some high-risk individuals cannot be ignored. This finding shared some similarities with that of Ruan et al.'s research,[26] which investigated the risk factors related to death of patients with COVID-19 using 150

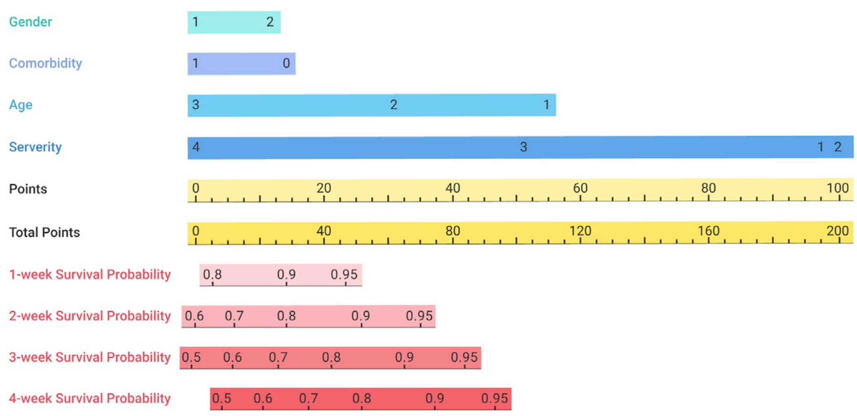

Figure 2. Predictive Model of Survival Nomogram for COVID-19 Patients Based on Four Key Predictors. Age was categorized to 1 (<45 years), 2 (45–60 years), and 3 ($\geq$60 years); Sex was divided into 0 (female) and 1 (male); Severity represents the severity of illness, where 1, 2, 3, and 4 represent mild, moderate, severe, and critical, respectively; Comorbidity represents existing chronic diseases, which was divided to 0 (no) and 1 (yes).

cases in Wuhan and suggested that the predictors of a fatal outcome in COVID-19 cases included age, underlying diseases, secondary infection, and increased inflammatory indicators in the blood. But, unlike our use of quantitative estimates of death probability, they simply compared the variables between death and non-death groups. Overall, more studies that utilize prediction models are warranted in future research to provide robust evidence. Previous studies[27] have shown that the accuracy of C-index is low at 0.50–0.70, medium at 0.71–0.90, and high at 0.90–1, so our estimated C-index (0.93) indicated a better prediction accuracy. Our calibration curves also showed a good model performance in the validation cohort between the nomogram prediction and actual observation for 1-, 2-, 3-, and 4-week survival.

In the context of the ongoing global pandemic of COVID-19 and the limited medical resources to deal with the outbreak,[28] it is urgently necessary to identify risk factors related to the case fatality of COVID-19 to enable clinicians to better tailor the treatment plans, better distribute limited medical resources, and improve the survival rate of COVID-19 patients. The predictive model can provide a scientific evidence base to inform the decision-making processes of government medical resource allocation. Based on the death probability of patients in different situations, allocation could be carried out using a hierarchical management to the most vulnerable groups.

Our study possessed a few advantages. We recruited 21,392 COVID-19 cases from Hubei, China, as our study subjects. The large sample size ensured that this study has a greater power to detect any differences that truly exist in the population. The study began in December 2019 and the follow-up was completed on March 18, 2020, which essentially covers the complete course of the COVID-19 outbreak in Hubei, China.

Several limitations should be noted. Selection bias was possible as we only recruited 21,392 participants out of the 70,233 cases in the study area, and our analysis observed a relatively higher CFR among excluded patients. There were 8,533 included patients who lacked complete laboratory testing results, which may lead to some degree of inaccuracy in our analysis. We only recruited the COVID-19 cases in Hubei Province, which might also result in selection bias. However, the bias would not be serious because approximately 75% of the cases in China occurred in Hubei. Data on clinical treatment and medical resources were not available for our analyses, which may potentially confound the estimate to some extent. Due to the inaccessibility of the data, we can only obtain laboratory test indicators of WBCs, lymphocytes, and neutrophils, and about 40% of participants had missing values for these laboratory testing. We suggest that more indicators that can reflect the physiological injury, such as C-reactive protein, D-dimer, and interleukin-6, should be included in future studies. In addition, because the detailed information of some variables cannot be obtained, there may be variable collinearity problems (such as hospital transfer and disease severity) in the process of model analysis. Only age, sex, severity of this disease, and comorbidity were included in the predictive model, as robust evidence suggested that the four indicators were closely related to the progression of COVID-19.[26,29–31] However, more confirmed variables should be included in future research. Finally, detailed information on the clinical course of patients was not available for this study, which limited the possibility of exploring the relationships between clinical progress outcomes and case fatality.

Our study confirms a wide range of risk factors to be determinants for COVID-19 fatality. These factors include demographic (older age and male), clinical (existing comorbidities, severe, and critical illness), and laboratory factors (decreased number of lymphocytes). The combination effects of age, sex, severity of the disease, and pre-existing diseases estimated from the predictive model could help identify the vulnerable populations and guide the allocation of medical resources.

MATERIALS AND METHODS

Study Participants

We constructed a retrospective cohort based on the National Notifiable Infectious Disease Reporting System and medical records of patients in Hubei Province, China. A total of 21,392 COVID-19 patients were selected to conduct a face-to-face interview. The inclusion criteria included that the patients were diagnosed with COVID-19 in Hubei Province and that their illness onset occurred between December 10, 2019, and February 27, 2020. Participants were excluded if they had missing information on important clinical variables (including fever, cough, and body temperature) and laboratory testing results (including the counts/percentages of white blood cells, lymphocytes, and neutrophils). To examine the representativeness of the included participants, we compared the demographic characteristics of these patients with remaining, excluded patients. The participants were recruited from December 10, 2019, to February 27, 2020, and their survival status was followed up until March 18, 2020.

This study was approved by the Ethics Review Committee of School of Public Health, Sun Yat-sen University (No.: 2020016). As the data collection was part of the infectious disease outbreak investigation, informed consent was waived.

Data Collection

The baseline demographic information was extracted from the National Notifiable Infectious Disease Reporting System. We collected additional information through face-to-face interviews, such as the comorbidity, occupation, and residence. The illness-related information was collected from hospital records, including the clinical symptoms, the date of symptom onset, the levels of the hospital of admittance, and laboratory testing results. Data for the analysis included age (<45, 45–60, and $\geq$60 years), sex (male and female), days between symptom onset and diagnosis (<7, 7–14, and $\geq$14 days), occupation (medical-related, service-related, office worker, home worker, and others), residence (local and migrant), hospitals level (grade I, grade II, and grade III),[32] severity of illness (mild/asymptomatic, moderate, severe, and critical), living close to a farmers' market (yes or no), area (Wuhan or non-Wuhan in Hubei), comorbidity (the existence of any of the following chronical diseases: hypertension, diabetes, kidney disease, liver disease, cardiovascular disease, respiratory disease, and immune system disease), and laboratory testing (counts of white blood cells, lymphocytes, and neutrophils).

The classification of the severity was based on a comprehensive analysis according to the guidelines for diagnosis and treatment of COVID-19 issued by Chinese National Health Commission.[33] Specifically, severe cases were defined as (1) respiratory rate $\geq$30 breaths/min, (2) oxygen saturation $\leq$93%, or (3) PaO_2/FiO_2 ratio $\leq$300 mm Hg. Critical severe cases were defined according to at least one of the following criteria: shock, respiratory failure requiring mechanical ventilation, combination with other organ failures, and admission to the intensive care unit. In this study, due to

the lack of detailed information about the patient's clinical process, disease severity was defined according to whether the patient experienced a severe or critical severe condition. For example, if a patient was admitted to hospital with mild symptoms but had severe conditions during the study period, they were defined as a severe patient.

Considering that a shortage of medical resources during the early stages of the pandemic may have been associated with a higher fatality rate of COVID-19, we included an indicator to reflect this temporal trend, with February 8 as the cutoff point according to the date of hospitalization of the patients; patients admitted to the hospital before February 8 were defined as 1 and those after February 8 as 0. The date of February 8 was selected for two reasons, first, two specifically designated hospitals (Huoshenshan Hospital and Leishenshan Hospital) and several Fangcang hospitals started to treat COVID-19 patients at around February 8; second, a number of medical workers and resources from other provinces arrived in Hubei around that time.

Laboratory testing data (white blood cell [WBC], lymphocytes, and neutrophils) were classified into three groups according to the normal ranges.[11] WBC was divided to low ($<4 \times 10^9$/L), reference (4–10 $\times 10^9$/L), and high levels ($>10 \times 10^9$/L); lymphocytes were divided to low ($<0.8 \times 10^9$/L), reference (0.8–4 $\times 10^9$/L), and high levels ($>4 \times 10^9$/L); lymphocyte percentage was divided to low (<20%), reference (20%–40%), and high levels (>40%); neutrophil percentage was grouped to low (<50%), reference (50%–70%), and high levels (>70%).

Case Definition

According to national guidelines, the COVID-19 cases were diagnosed using four different definitions in Hubei Province. A confirmed case was defined as a positive result of high-throughput sequencing or real-time reverse transcription polymerase chain reaction (RT-PCR) in throat swab specimens.[33] To identify SARS-CoV-2 infection, throat swab samples were obtained from all patients at the time of hospital admission.[11] A clinically diagnosed case was defined based on symptoms, exposure, and pulmonary imaging characteristics, but no nucleic acid test was conducted. Positive chest computed tomography findings were recommended as the key evidence for clinically confirmed cases by Chinese management guidelines for COVID-19 (version 7.0).[33] However, due to the limited detection capacity at the outbreak period, some individuals were also diagnosed as suspected cases. A suspected patient was defined based on clinical symptoms and exposure history only. An asymptomatic case was diagnosed by positive viral nucleic acid test result without typical symptoms, including fever, dry cough, and fatigue.[34] Among the 21,392 cases, 18,470 were confirmed COVID-19 cases, 2,015 were clinically diagnosed cases, 865 were suspected cases, and 42 were asymptomatic cases.

Statistical Analysis

We summarized the distribution of continuous variables by median and interquartile range values. The differences of continuous variables across deceased and living groups were examined by Student's t tests, and categorical variables were compared with chi-square test and Fisher's exact test.

Univariate and multivariate Cox proportional hazards models were fitted to quantify the associations of potential risk factors with the fatality of COVID-19.[35,36] The indicator variable "Period" was also included in the multivariate models to control the impact of medical resources at different stages of the epidemic. Variables with a $p < 0.10$ in univariate analysis were included in the multivariate models. The survival time used in Cox analyses was defined by days from the symptom onset date to date of death or to the last day of follow-up on March 18, 2020, whichever came first. The associations were shown as hazard ratios (HR) and 95% confidence intervals (CI). We also plotted survival curves according to age, sex, severity of illness, and comorbidity. The curves showed the survival probability with the days from symptom onset, which graphically demonstrated the differences between different strata of the predictors.[37]

Sensitivity analyses were conducted to test the robustness of the findings. We performed one analysis by restricting the participants to the confirmed COVID-19 cases. We also compared some demographic factors among the included (n = 21,392) and excluded participants (n = 48,841) and assessed the associations of these factors with CFR in the two groups.

Constructing a Nomogram for Prediction

A predictive model was then constructed based on the nomogram approach. A nomogram is a reliable tool to quantify survival likelihood of one individual by incorporating the important factors affecting the prognosis of the disease and has been widely used to evaluate prognostic factors in cancer patients.[38,39] The nomogram is based on the contribution of each factor to the outcome in the model, from which an influence score is given, and then the total score of each individual is calculated to predict the survival probability of each individual.[39,40] For the easy application purpose, we included four main variables for formulating a nomogram in this study, including sex, age, comorbidity, and the severity of the disease in the predictive model, with the aim to identify which combination would have the greatest impact on the death of a COVID-19 patient.

The concordance index (C-index) represents the performance of model prediction. It varies from 0.5 to 1.0, with 0.5 showing random chance and 1.0 indicating a good ability to discriminate the outcome.[41] To assess the validation of the model, we adopted a random sampling method to select 80% of samples as a training cohort dataset and 20% of samples as a validation cohort dataset. The nomogram was subjected to 1,000 bootstrap resamples for internal validation, and calibration of the nomogram for 1-, 2-, 3-, and 4-week survival was performed by comparing the predicted survival with the observed survival after correcting for bias.

All statistical analyses were performed using R 3.4.3, with $p < 0.05$ (two-sided) as statistically significant.

REFERENCES

1. Rothan, H., and Siddappa, N. (2020). The epidemiology and pathogenesis of coronavirus disease (COVID-19) outbreak. J. Autoimmun. *109*, 102433.
2. Liu, K., Ai, S., Song, S., Zhu, G., Tian, F., Li, H., Gao, Y., Wu, Y., Zhang, S., Shao, Z., et al. (2020). Population movement, city closure in Wuhan and geographical expansion of the 2019-nCoV pneumonia infection in China in January 2020. Clin. Infect. Dis. https://doi.org/10.1093/cid/ciaa422.
3. Mahase, E. (2020). Coronavirus: Covid-19 has killed more people than SARS and MERS combined, despite lower case fatality rate. BMJ *368*, m641.
4. Onder, G., Rezza, G., and Brusaferro, S. (2020). Case-fatality rate and characteristics of patients dying in relation to COVID-19 in Italy. JAMA. https://doi.org/10.1001/jama.2020.4683.
5. Zhou, F., Yu, T., Du, R., Fan, G., Liu, Y., Liu, Z., Xiang, J., Wang, Y., Song, B., Gu, X., et al. (2020). Clinical course and risk factors for mortality of adult inpatients with COVID-19 in Wuhan, China: a retrospective cohort study. Lancet *395*, 1054–1062.
6. Chu, J., Yang, N., Wei, Y., Yue, H., Zhang, F., Zhao, J., He, L., Sheng, G., Chen, P., Li, G., et al. (2020). Clinical characteristics of 54 medical staff with COVID-19: a retrospective study in a single center in Wuhan, China. J. Med. Virol. *92* (7), 807–813.
7. Deng, Y., Liu, W., Liu, K., Fang, Y.Y., Shang, J., Zhou, L., Wang, K., Leng, F., Wei, S., Chen, L., and Liu, H.G. (2020). Clinical characteristics of fatal and recovered cases of coronavirus disease 2019 (COVID-19) in Wuhan, China: a retrospective study. Chin Med. J. (Engl). *133*, 1261–1267.
8. Du, Y., Tu, L., Zhu, P., Mu, M., Wang, R., Yang, P., Wang, X., Hu, C., Ping, R., Hu, P., et al. (2020). Clinical features of 85 fatal cases of COVID-19 from Wuhan: a retrospective observational study. Am. J. Respir. Crit. Care Med. *201*, 1372–1379.
9. Chen, C., Wang, H., Liang, Z., Peng, L., Zhao, F., Yang, L., Cao, M., Wu, W., Jiang, X., Zhang, P., et al. (2020). Predicting illness severity and short-term outcomes of COVID-19: a retrospective cohort study in China. Innovation *1*, 100007.
10. Liu, W., Tao, Z.W., Lei, W., Ming-Li, Y., Kui, L., Ling, Z., Wei, S., Deng, Y., Liu, J., Liu, H.G., et al. (2020). Analysis of factors associated with disease outcomes in hospitalized patients with 2019 novel coronavirus disease. Chin. Med. J. (Engl). 133, https://doi.org/10.1097/CM9.0000000000000775.
11. Wu, C., Chen, X., Cai, Y., Xia, Ja, Zhou, X., Xu, S., Huang, H., Zhang, L., Zhou, X., Du, C., et al. (2020). Risk factors associated with acute respiratory distress syndrome and death in patients with coronavirus disease 2019 pneumonia in Wuhan, China. JAMA Intern. Med. e200994, https://doi.org/10.1001/jamainternmed.2020.0994.
12. Choi, K.W., Chau, T.N., Tsang, O., Tso, E., Chiu, M.C., Tong, W.L., Lee, P.O., Ng, T.K., Ng, W.F., Lee, K.C., et al. (2003). Outcomes and prognostic factors in 267 patients with severe acute respiratory syndrome in Hong Kong. Ann. Intern. Med. *139*, 715–723.
13. Jin, J.-M., Bai, P., He, W., Wu, F., Liu, X.-F., Han, D.-M., Liu, S., and Yang, J.K. (2020). Gender differences in patients with COVID-19: focus on severity and mortality. Front. Public Health *8*.
14. Liu, J., Zheng, X., Tong, Q., Li, W., Wang, B., Sutter, K., Trilling, M., Lu, M., Dittmer, U., Yang, D., et al. (2020). Overlapping and discrete aspects of the pathology and pathogenesis of the emerging human pathogenic coronaviruses SARS-CoV, MERS-CoV, and 2019-nCoV. J. Med. Virol. *92*, 491–494.
15. de Wit, E., van Doremalen, N., Falzarano, D., and Munster, V.J. (2016). SARS and MERS: recent insights into emerging coronaviruses. Nat. Rev. Microbiol. *14*, 523–534.
16. Channappanavar, R., Fett, C., Mack, M., and Ten Eyck, P.P. (2017). Sex-based differences in susceptibility to severe acute respiratory syndrome coronavirus infection. J. Immunol. *198*, 4046–4053.
17. Chen, N., Zhou, M., Dong, X., Qu, J., Gong, F., Han, Y., Qiu, Y., Wang, J., Liu, Y., Wei, Y., et al. (2020). Epidemiological and clinical characteristics of 99 cases of 2019 novel coronavirus pneumonia in Wuhan, China: a descriptive study. Lancet *395*, 507–513.
18. Guan, W.-j., Ni, Z.-y., Hu, Y., Liang, W.-h., Ou, C.-q., He, J.-x., Liu, L., Shan, H., Lei, C.L., Hui, D.S.C., et al. (2020). Clinical characteristics of coronavirus disease 2019 in China. N. Engl. J. Med. *382*, 1708–1720.
19. Huang, R., Zhu, L., Xue, L., Liu, L., Yan, X., Wang, J., Zhang, B., Xu, T., Ji, F., Zhao, Y., et al. (2020). Clinical findings of patients with coronavirus disease 2019 in Jiangsu Province, China: a retrospective, multi-center study. PLoS Negl. Trop. Dis. *14*, e0008280.
20. Zhang, J.J., Dong, X., Cao, Y.Y., Yuan, Y.D., Yang, Y.B., Yan, Y.Q., Akdis, C.A., and Gao, Y.D. (2020). Clinical characteristics of 140 patients infected with SARS-CoV-2 in Wuhan, China. Allergy. https://doi.org/10.1111/all.14238.

21. Zhou, P., Yang, X.L., Wang, X.G., Hu, B., Zhang, L., Zhang, W., Si, H.R., Zhu, Y., Li, B., Huang, C.L., et al. (2020). A pneumonia outbreak associated with a new coronavirus of probable bat origin. Nature *579*, 270–273.
22. Hamming, I., Timens, W., Bulthuis, M.L., Lely, A.T., Navis, G., and van Goor, H. (2004). Tissue distribution of ACE2 protein, the functional receptor for SARS coronavirus. A first step in understanding SARS pathogenesis. J. Pathol. *203*, 631–637.
23. Murthy, S., Gomersall, C.D., and Fowler, R.A. (2020). Care for critically ill patients with COVID-19. JAMA *323*, 1499–1500.
24. Ye, G., Lin, H., Chen, S., Wang, S., Zeng, Z., Wang, W., Zhang, S., Rebmann, T., Li, Y., Pan, Z., et al. (2020). Environmental contamination of SARS-CoV-2 in healthcare premises. J. Infect. https://doi.org/10.1016/j.jinf.2020.04.034.
25. Celli, B., and Fabbri, L. (2020). Urgent need of a management plan for survivors of COVID-19. Eur. Respir. J. *55*, 2000764.
26. Ruan, Q., and Yang, K. (2020). Clinical predictors of mortality due to COVID-19 based on an analysis of data of 150 patients from Wuhan, China. Intens. Care Med. *46*, 846–848.
27. Yang, X.G., Feng, J.T., Wang, F., He, X., Zhang, H., Yang, L., Zhang, H.R., and Hu, Y.C. (2019). Development and validation of a prognostic nomogram for the overall survival of patients living with spinal metastases. J. Neurooncol. *145*, 167–176.
28. PLOS Medicine Editors (2020). Pandemic responses: planning to neutralize SARS-CoV-2 and prepare for future outbreaks. PLoS Med. *17*, e1003123.
29. Chen, T., Dai, Z., Mo, P., Li, X., Ma, Z., Song, S., Chen, X., Luo, M., Liang, K., Gao, S., et al. (2020). Clinical characteristics and outcomes of older patients with coronavirus disease 2019 (COVID-19) in Wuhan, China (2019): a single-centered, retrospective study. J. Gerontol. A. Biol. Sci. Med. Sci. https://doi.org/10.1093/gerona/glaa089.
30. Li, X., Xu, S., Yu, M., Wang, K., Tao, Y., Zhou, Y., Shi, J., Zhou, M., Wu, B., Yang, Z., et al. (2020). Risk factors for severity and mortality in adult COVID-19 inpatients in Wuhan. J. Allergy Clin. Immunol. https://doi.org/10.1016/j.jaci.2020.04.006.
31. Liao, J., Fan, S., Chen, J., Wu, J., Xu, S., Guo, Y., Li, C., Zhang, X., Wu, C., Mou, H., et al. (2020). Epidemiological and clinical characteristics of COVID-19 in adolescents and young adults. Innovation *1*, 100001.
32. Cai, M., Liu, E., Tao, H., Qian, Z., Lin, X., and Cheng, Z. (2018). Does level of hospital matter? A study of mortality of acute myocardial infarction patients in Shanxi, China. Am. J. Med. Qual. *33* (2), 185–192.
33. National Health Commission (2020). Chinese management guideline for COVID-19 (version 7.0). March 3, 2020. http://www.nhc.gov.cn/yzygj/s7653p/202003/46c9294a7dfe4cef80dc7f5912eb1989/files/ce3e6945832a438eaae415350a8ce964.pdf.
34. Wu, Z., and McGoogan, J.M. (2020). Characteristics of and important lessons from the coronavirus disease 2019 (COVID-19) outbreak in China: summary of a report of 72 314 cases from the Chinese center for disease control and prevention. JAMA *323*, 1239–1242.
35. Jang, S.Y., Cha, Y.H., Kim, K.J., Kim, H.Y., and Choy, W.S. (2020). The effect of surgery type on mortality in elderly patients with pertrochanteric femoral fracture: a Korean nationwide cohort study. Asian J. Surg. *43* (4), 550–556.
36. Jhee, J.H., and Joo, Y.S. (2020). Intensity of statin therapy and renal outcome in chronic kidney disease: results from the Korean cohort study for outcome in patients with chronic kidney disease. Kidney Res. Clin. Pract. *39* (1), 93–102.
37. Kim, J., Hong, J.Y., Kim, S.T., Park, S.H., Jekal, S.Y., Choi, J.S., Chang, D.K., Kang, W.K., Seo, S.W., and Lee, J. (2020). Clinical scoring system for the prediction of survival of patients with advanced gastric cancer. ESMO Open *5* (2), e000670.
38. Wierda, W.G., O'Brien, S., Wang, X., Faderl, S., Ferrajoli, A., Do, K.A., Cortes, J., Thomas, D., Garcia-Manero, G., Koller, C., et al. (2007). Prognostic nomogram and index for overall survival in previously untreated patients with chronic lymphocytic leukemia. Blood *109*, 4679–4685.
39. Tang, L.Q., Li, C.F., Li, J., Chen, W.H., Chen, Q.Y., Yuan, L.X., Lai, X.P., He, Y., Xu, Y.X., Hu, D.P., et al. (2015). Establishment and validation of prognostic nomograms for endemic nasopharyngeal carcinoma. J. Natl. Cancer Inst. *108*, djv291.
40. Li, H., and Wang, Z. (2020). Development and validation of a nomogram for predicting cancer-specific survival of surgical resected stage I-II adenosquamous carcinoma of the lung. J. Surg. Oncol. *121*, 1027–1035.
41. Wang, Y., Li, J., Xia, Y., Gong, R., Wang, K., Yan, Z., Wan, X., Liu, G., Wu, D., Shi, L., et al. (2013). Prognostic nomogram for intrahepatic cholangiocarcinoma after partial hepatectomy. J. Clin. Oncol. *31*, 1188–1195.

ACKNOWLEDGMENTS

We thank all the staff involved in the field survey and the anonymous reviewers for the valuable comments. We appreciate the support provided by the National Natural Science Foundation of China (82041021).

AUTHOR CONTRIBUTIONS

M.L. and Hualiang Lin planned the study, analyzed and interpreted the data , and revised the manuscript. J.C. and R.W. was responsible for data collection and validation, and data analysis and interpretation, and revised the manuscript. S.A. and S.Z. contributed to data analysis and result interpretation, and drafted the manuscript. Z.Q. revised the manuscript and interpreted the findings. Y.Z., Y.W., L.C., F.T., and Huan Li participated in data analysis, interpretation, and manuscript revision. All the authors have given final approval to the manuscript to be published and agreed to be responsible for all aspects of the manuscript.

DECLARATION OF INTERESTS

The authors declare no competing interests.

SUPPLEMENTAL INFORMATION

Supplemental Information can be found online at https://doi.org/10.1016/j.xinn.2020.100022.

LEAD CONTACT WEBSITE

http://sph.sysu.edu.cn/teacher/361.

XINN, Volume *1*

Supplemental Information

Predictive Model and Risk Factors for Case Fatality of COVID-19: A Cohort of 21,392 Cases in Hubei, China

Ran Wu, Siqi Ai, Jing Cai, Shiyu Zhang, Zhengmin (Min) Qian, Yunquan Zhang, Yinglin Wu, Lan Chen, Fei Tian, Huan Li, Mingyan Li, and Hualiang Lin

Supplementary Materials

Table S1. Point assignment and one-, two- three- and four-week death probability of a male patient.

Comorbidity	Age group	Severity	Total points	Death probability (%)			
				Week 1	Week 2	Week 3	Week 4
No	< 45	Mild	163	1	1	2	3
		Moderate	166	1	1	2	3
		Severe	116	1	2	3	4
		Critical	66	2	5	7	13
	45-60	Mild	145	1	1	2	3
		Moderate	148	1	1	2	3
		Severe	98	2	4	4	5
		Critical	48	4	10	15	20
	≥ 60	Mild	110	1	2	3	4
		Moderate	113	1	2	3	4
		Severe	63	2	7	9	13
		Critical	13	15	26	36	44
Yes	< 45	Mild	150	1	1	2	3
		Moderate	153	1	1	2	3
		Severe	103	2	3	4	5
		Critical	53	3	8	13	16
	45-60	Mild	132	1	1	2	3
		Moderate	135	1	1	2	3
		Severe	85	1	3	4	6
		Critical	35	7	15	21	27
	≥ 60	Mild	97	2	4	4	5
		Moderate	100	2	4	4	5

		Severe	50	4	9	15	20
		Critical	0	21	36	46	54

Table S2. Point assignment and one-, two- three- and four-week death probability of a female patient.

Comorbidity	Age group	Severity	Total points	Death probability (%)			
				Week 1	Week 2	Week 3	Week 4
No	< 45	Mild	173	1	1	2	3
		Moderate	176	1	1	2	3
		Severe	126	1	1	2	3
		Critical	76	2	4	7	8
	45-60	Mild	155	1	1	2	3
		Moderate	158	1	1	2	3
		Severe	108	2	4	3	4
		Critical	58	3	8	11	15
	≥ 60	Mild	120	1	2	3	4
		Moderate	123	1	2	3	4
		Severe	73	2	5	8	9
		Critical	23	11	20	30	36
Yes	< 45	Mild	160	1	1	2	3
		Moderate	163	1	1	2	3
		Severe	113	2	3	4	5
		Critical	63	2	6	10	14
	45-60	Mild	142	1	1	2	3
		Moderate	145	1	1	2	3
		Severe	95	2	4	5	5
		Critical	45	5	11	16	22

≥ 60	Mild	107	2	4	4	5
	Moderate	110	2	4	4	5
	Severe	60	3	6	10	15
	Critical	10	16	30	40	46

Table S3. The comparison of general characteristics of the included (n=21,392) and excluded participants (n=48,841) in Hubei, China.

**Variable**	**Included (n=21,392)**	**Excluded (n=48,841)**	***P* value**
**Sex**			<0.05
Male	11,099 (51.88)	23,295 (47.69)	
Female	10,293 (48.12)	25,548 (52.31)	
**Age**			<0.05
< 45 years	8,127 (37.99)	14,445 (29.57)	
45-60 years	7,641 (35.72)	15,046 (30.80)	
≥ 60 years	5,624 (26.29)	19,352 (39.62)	
**Death**			0.21
Deceased	1,020 (4.77)	2,441 (5.00)	
Alive	20,372 (95.23)	46,402 (95.00)	

Table S4. Sensitivity analysis by restricting the participants to the confirmed cases (n=18,470).

	**Univariable model**		**Multivariable model**	
	**HR (95% CI)**	***P* value**	**HR (95% CI)**	***P* value**
**Sex**				
Female	1.00		1.00	
Male	1.67 (1.46, 1.92)	<0.001	1.34 (1.17, 1.53)	<0.01

**Age group**				
< 45 years	1.00		1.00	
45-60 years	3.26 (2.37, 4.48)	<0.001	2.28 (1.65, 3.15)	<0.001
≥ 60 years	21.98 (16.50, 29.27)	<0.001	7.04 (5.14, 9.62)	<0.001
**Comorbidity**				
No	1.00		1.00	
Yes	4.01 (3.52, 4.56)	<0.001	1.43 (1.25, 1.64)	<0.001
**Severity**				
Mild/asymptomatic	1.00		1.00	
Moderate	1.04 (0.73, 1.47)	>0.05	1.08 (0.76, 1.53)	>0.05
Severe	12.43 (9.36, 16.51)	<0.001	7.86 (5.90, 10.47)	<0.001
Critical	76.75 (58.50, 100.69)	<0.001	42.57 (32.26, 56.19)	<0.001

Table S5. Comparison of the effects of the general demographic factors using the included (n=21,392) and excluded participants (n=48,841).

	**Included participants**		**Excluded participants**	
	**HR (95% CI)**	***P* value**	**HR (95% CI)**	***P* value**
**Sex**				
Female	1.00		1.00	
Male	1.72 (1.51, 1.95)	<0.001	1.91 (1.76, 2.08)	<0.001
**Age group**				
< 45 years	1.00		1.00	
45-60 years	3.56 (2.62, 4.83)	<0.001	4.27 (3.34, 5.46)	<0.001
≥ 60 years	21.36 (16.20, 28.16)	<0.001	19.27 (15.34, 24.19)	<0.001
**Residence**				
Local	1.00		1.00	
Migrant	1.11 (0.96, 1.28)	>0.05	1.33(1.23, 1.45)	<0.001

**Area**				
Hubei-other cities	1.00		1.00	
Hubei-Wuhan	1.53 (1.44, 1.62)		1.62 (1.49, 1.76)	
**Period**				
Before Feb. 8	1.00		1.00	
After Feb. 8	0.44 (0.37, 0.52)	<0.001	0.53 (0.49, 0.57)	<0.001

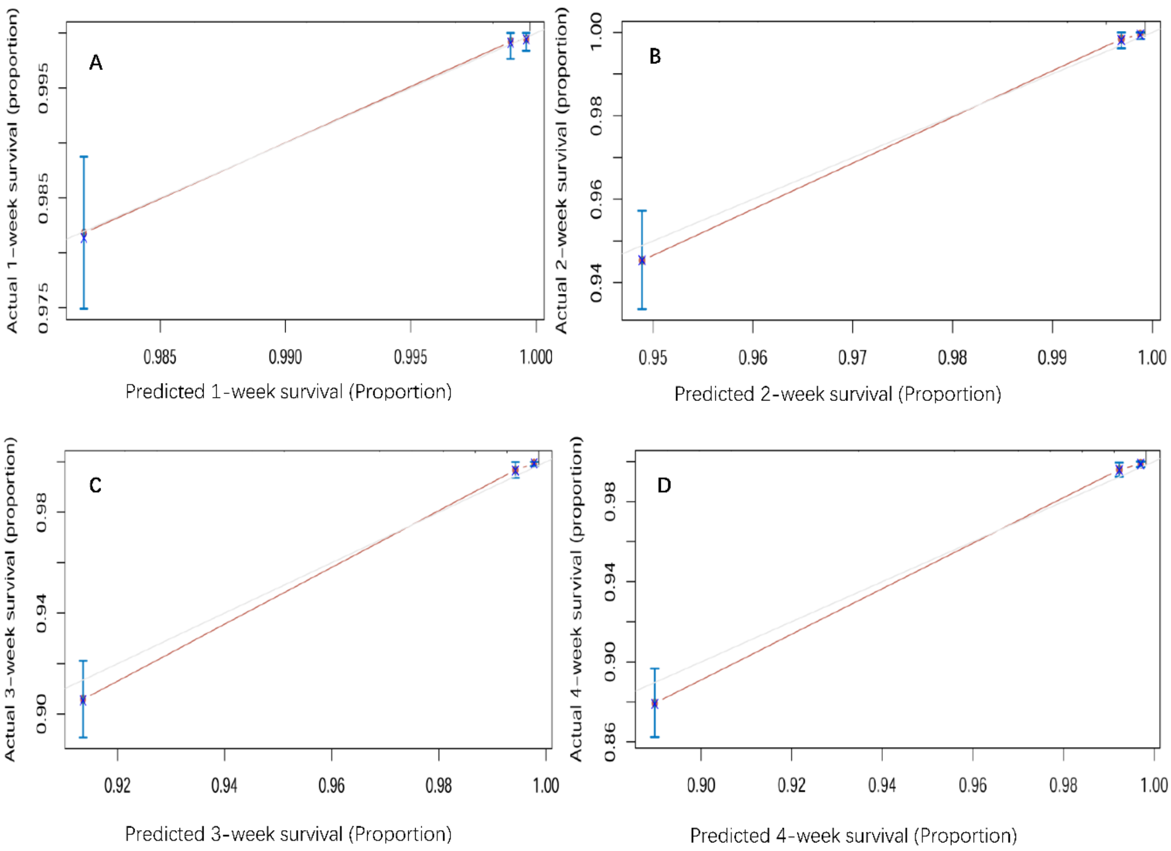

Figure S1. The calibration curve for predicting patient survival in (A) one-week and, (B) two-week, (C) three-week and (D) four-week in the validation cohort.

Note: Nomogram-predicted survival probability of patients with COVID-19 is plotted on the x-axis; actual survival probability is plotted on the y-axis.
